# Multimodal MRI Evaluation of the MitoPark Mouse Model of Parkinson’s Disease

**DOI:** 10.1371/journal.pone.0151884

**Published:** 2016-03-22

**Authors:** Linlin Cong, Eric R. Muir, Cang Chen, Yusheng Qian, Jingwei Liu, K. C. Biju, Robert A. Clark, Senlin Li, Timothy Q. Duong

**Affiliations:** 1 Research Imaging Institute, University of Texas Health Science Center, San Antonio, Texas, United States of America; 2 Graduate School of Biomedical Science, University of Texas at San Antonio, San Antonio, Texas, United States of America; 3 Department of Ophthalmology, University of Texas Health Science Center, San Antonio, Texas, United States of America; 4 Department of Medicine, University of Texas Health Science Center, San Antonio, Texas, United States of America; 5 South Texas Veterans Health Care System, Department of Veterans Affairs, San Antonio, Texas, United States of America; Istituto Italiano di Tecnologia, ITALY

## Abstract

The MitoPark mouse, a relatively new genetic model of Parkinson’s disease (PD), has a dopaminergic neuron-specific knock-out that inactivates the mitochondrial transcription factor A (*Tfam*), a protein essential for mitochondrial DNA expression and maintenance. This study used multimodal MRI to characterize the neuroanatomical correlates of PD-related deficits in MitoPark mice, along with functional behavioral tests. Compared with age-matched wild-type animals, MitoPark mice at 30 weeks showed: *i)* reduced whole-brain volume and increased ventricular volume, indicative of brain atrophy, *ii)* reduced transverse relaxation time (T_2_*) of the substantia nigra and striatum, suggestive of abnormal iron accumulation, *iii)* reduced apparent diffusion coefficient in the substantia nigra, suggestive of neuronal loss, *iv)* reduced fractional anisotropy in the corpus callosum and substantia nigra, indicative of white-matter damages, *v)* cerebral blood flow was not significantly affected, and *vi)* reduced motor activity in open-field tests, reduced memory in novel object recognition tests, as well as decreased mobility in tail suspension tests, an indication of depression. In sum, MitoPark mice recapitulate changes in many MRI parameters reported in PD patients. Multimodal MRI may prove useful for evaluating neuroanatomical correlates of PD pathophysiology in MitoPark mice, and for longitudinally monitoring disease progression and therapeutic interventions for PD.

## Introduction

Parkinson's disease (PD) the second most common neurodegenerative disease, is characterized primarily by death of dopaminergic (DA) neurons in the substantia nigra [1]. The specific etiology of PD remains unknown. Current PD treatments are primarily based on pharmacological replacement of dopamine to treat motor symptoms, providing only symptomatic relief for a few years in the early stages of PD [2]. Animal models provide an important means to investigate PD etiology, pathology, and therapeutic approaches. They include acute toxin models, such as 6-hydroxydopamine (6-OHDA) or 1-methyl-4phenyl-1,2,3,6-tertrahydropyridine (MPTP) [3], as well as genetic models, such as α-synuclein, PINK1, Parkin and LRRK2 alterations [4]. Given the likelihood of multiple contributing etiologic factors and pathological processes in PD, as well as heterogeneity in the expression and progression of the clinical manifestations of the disease, it is unlikely that a single animal model can mimic all features of human PD [5]. Some toxin models are well established and have been widely used to test treatments of motor symptoms, but they do not replicate the progressive nature and the pathological accumulation of neuronal inclusions seen in human PD. Genetic models overcome some drawbacks of acute models and can recapitulate specific features of PD, but generally none are able to replicate the progressive DA neuron loss associated with PD initiated by mitochondrial dysfunction, which has been linked to familial Parkinsonism [4, 6–9].

The MitoPark mouse, a relatively new genetic model of PD, has a dopaminergic neuron-specific knock-out inactivating mitochondrial transcription factor A (*Tfam*), a protein essential for mitochondrial DNA expression and maintenance [10]. This causes respiratory chain deficiency due to reduced mitochondrial DNA expression and mitochondrial dysfunction specifically in DA neurons [10]. MitoPark mice begin developing small cytoplasmic aggregates in midbrain dopamine neurons starting at 6 weeks of age [10], neuron loss starting at 12 weeks, and progressive motor symptoms starting at 14 weeks [11]. The MitoPark model replicates several essential features of PD, including adult onset of DA neuron loss, slow progressive neurodegeneration, formation of intraneuronal inclusions albeit without α-synuclein, responsiveness to levodopa (L-DOPA) treatment [10, 12, 13], and non-motor deficits, such as early cognitive impairment and signs of depression-like behavior [11, 12].

Magnetic resonance imaging (MRI) has been used to study disease pathology and progression in PD. The advantages of MRI are that it is non-invasive and provides multiple structural, physiological and functional data at the whole brain level. Structural MRI of PD shows enlarged ventricular volume and atrophy in the substantia nigra (SN) and other brain regions [14–17]. Diffusion tensor imaging shows changes in apparent diffusion coefficient (ADC) in various brain structures, indicative of neurodegeneration, and changes in fractional anisotropy (FA) of white matter structures, indicative of myelin and/or axonal degeneration [18, 19]. Some studies have reported reduced cerebral blood flow (CBF) MRI in different brain regions [20, 21]. T2* MRI of PD patients shows changes in the SN, suggesting increased iron accumulation [17, 22].

Although MitoPark mice have been studied using behavioral and histological techniques [10, 11, 23], MRI has not to our knowledge been used for non-invasive characterization of this model. These quantitative MRI parameters could allow longitudinal evaluation of the onset and progression of neurodegeneration and of therapeutic efficacy in MitoPark mice. Furthermore, comparable MRI findings from human patients and MitoPark mice could provide a means to explore the correlation of translational MRI exams with underlying histopathological progression. The goal of this study was to apply multi-parametric quantitative MRI to investigate changes in brain volume, transverse relaxation time (T2*), diffusion, and CBF in MitoPark mice compared with aged-matched wild-type animals. Comparisons were also made with functional behavioral tests.

## Materials and Methods

### Animals

All experimental procedures were approved by the Institutional Animal Care and Use Committee at the University of Texas Health Science Center, San Antonio. MitoPark mice (DAT^+/cre^-Tfam^loxP/loxP^) were previously generated [10], and we obtained breeding pairs from Dr. Nils-Göran Larsson (Max Planck Institute for Biology of Aging, Cologne, Germany). To generate experimental mice and littermate controls, MitoPark mice (on a C57BL/6 background) were backcrossed to C57BL/6J (The Jackson Laboratory, Bar Harbor, ME), and offspring were selectively mated to generate double heterozygous males (DAT^+/cre^-Tfam^+/loxP^) and homozygous floxed Tfam females (DAT^+/+^-Tfam^loxP/loxP^). Offspring from this breeding combination exhibit a 25% MitoPark genotype (DAT^*+/cre*^; Tfam^*loxP/loxP*^) at expected Mendelian inheritance probabilities. Genotyping was performed as describe before [10, 11]. Mice were group-housed with the same gender with *ad libitum* access to food and water. The room temperature was maintained at 26°C, with a 12-hour light on/12-hour light off cycle. MRI and behavioral tests were performed on separate groups of animals.

### MRI

MRI scans were performed on 6 MitoPark mice (3 females and 3 males) and 9 male wild-type C57BL/6J control mice (Jackson Laboratory) at 30 weeks of age. Mice were anesthetized with 5% isoflurane and maintained with 1.1%-1.5% isoflurane under spontaneous breathing conditions. MRI was performed in a 7 T, 30 cm magnet with a 1500 mT/m gradient insert (Bruker, Billerica, MA, USA). The animal was secured in a custom-made stereotaxic holder with ear and tooth bars. A transceiver surface coil was placed on the top of the head for imaging, and a labeling coil was placed under the heart for arterial spin labeling (ASL) [24]. Rectal temperature was maintained at 37.0±0.5°C. Respiration rate was monitored with an MR-compatible small animal monitoring and gating system (SA Instruments, Inc, Stony Brook, NY). Heart rate and arterial oxygen saturation were also monitored using a MouseOx system (Starr Life Sciences Corp., Oakmont, PA) and maintained within normal physiological ranges.

CBF images were acquired using continuous ASL with a 2.2 s labeling pulse to the labeling coil in the presence of a 20 mT/m gradient and a 350 ms post-label delay. Paired images with and without labeling were acquired in an interleaved fashion. MRI was acquired using a single-shot, gradient-echo, echo planar imaging (EPI) sequence with 250 kHz spectral width, 2.9 s repetition time (TR) and 8 ms echo time (TE). The field of view was 12.8x12.8 mm with a matrix size of 64x64. Nine coronal slices were acquired with 1 mm thickness. Two hundred repetitions were acquired.

Diffusion tensor imaging (DTI) was obtained using a spin-echo EPI sequence with 250 kHz spectral width, diffusion gradient separation Δ of 7.562 ms, diffusion gradient duration δ of 2 ms, and a b value of 1,200 s/mm^2^ applied in 30 diffusion directions. An image with b = 0 s/mm^2^ was also acquired. TR was 3 s and TE was 32 ms. The field of view was 12.8x12.8 mm with a matrix size of 64x64. Eleven coronal slices were acquired with 1 mm thickness. Eight averages were acquired.

T2* maps were calculated from a 3D multiple gradient echo sequence with a spectral width of 59 kHz. Five gradient echoes were acquired with the first TE = 2.4 ms and an inter-echo time of 3.3 ms, and only echoes during the positive gradient lobes were acquired. The TR was 40 ms. The field of view was 12.8x12.8x8 mm with an acquired matrix size of 86x86x40, reconstructed to 128x128x80. Six averages were acquired.

No motion correction was needed nor applied. The CBF, FA, ADC and T2* maps were calculated using codes written Matlab (MathWorks, Natick, MA) [25, 26]. CBF in units of mL/g/min was calculated using $CBF=\frac{\lambda }{T_{1}}\frac{S_{c}-S_{L}}{S_{c}+(2\alpha -1)S_{L}}$ where S_C_ is the control image signal intensity and S_L_ is the labeled image signal intensity, λ of 0.9 is the water tissue-blood partition coefficient [27], T1 of 1.8 s is the average whole brain spin-lattice relaxation constant at 7 T [28], and α of 0.75 is the labeling efficiency [24]. T2* maps were calculated using linear regression to fit ln(S_i_) = ln(S_0_)–(TE_i_/T2*) where S_i_ is the signal intensity at echo time TE_i_ and S_0_ is the equilibrium magnetization. DTI images were analyzed to calculate $ADC=\frac{\lambda_{1}+\lambda_{2}+\lambda_{3}}{3}$, and $FA=\frac{\sqrt{3[{\left(\lambda_{1}-ADC\right)}^{2}+{\left(\lambda_{2}-ADC\right)}^{2}+{\left(\lambda_{3}-ADC\right)}^{2}]}}{\sqrt{{2[(\lambda_{1})}^{2}+{\left(\lambda_{2}\right)}^{2}+{\left(\lambda_{3}\right)}^{2}}]}$ where λ_1_, λ_2_, and λ_3_ are the eigenvalues of the diffusion tensor [29].

Brain volume was measured from T2*-weighted images, and ventricular volumes were obtained from ADC maps. The brain (excluding the olfactory bulb and cerebellum) was manually segmented based on the T2*-weighted images. The ventricles were then segmented by thresholding ADC values above three standard deviations of the mean. The ventricular volume and the brain tissue volume excluding the ventricular volume were tabulated.

For quantitation of MRI parameters, bilateral ROIs, including the whole brain, striatum, substantia nigra (SN), motor cortex, thalamus, hippocampus, auditory-visual cortex, sensory cortex, and corpus callosum (CC), were drawn manually on each animal using the Paxinos mouse atlas for reference [30]. ROIs that are involved in the motor network and known to be affected in MitoPark mice were chosen including the substantia nigra, striatum, and motor cortex [10]. The thalamus, which has connections with the basal ganglia and motor cortex, and hippocampus were also analyzed, both of which are reported to undergo atrophy in PD [31], although a previous study found that dopamine levels remain normal in the hippocampus of MitoPark mice [10]. A few cortical regions which are not implicated in motor activity were also analyzed to determine the extent of potential changes in the brain, including the sensory cortex, which is adjacent to the motor cortex, and the auditory-visual cortex which is relatively far from the motor cortex. FA of the corpus callosum was also analyzed as it is the most distinct white matter structure in rodents and is not expected to be directly affected by dopamine neuron loss.

The same ROIs were applied for CBF and DTI images which were acquired with EPI and with the same geometry, whereas ROIs were drawn separately for T2* maps. T2* maps from 3 wild-type and from 1 MitoPark mice were excluded due to the presence of visible fringe artifacts. Additionally, data from select ROIs were excluded in two other MitoPark and two controls due to localized artifacts (see S1 Dataset for the complete data set).

### Behavioral tests

Motor Activity: Horizontal and vertical locomotor activities were measured using the Photobeam Activity System (San Diego, CA) according to the manufacturer’s protocol. Animals were acclimatized to the testing room for at least 1 h prior to the start of testing. The mice were individually placed in a clear polycarbonate testing cage (18 cm W x 29 cm L x 12 cm H) with approximately 0.5 cm of corncob bedding lining the floor. Their horizontal and vertical locomotor activities were recorded for 60 min and assessed at 10-min time intervals. Motor activity was measured in 8 male MitoPark mice and 13 male wild-type littermate controls at 30 weeks of age.

Novel Object Recognition Test: To minimize the effects of stress, mice were transported to the testing room (under dim overhead white lighting) and gently handled for two consecutive days. On the third and fourth day, mice were placed in the empty training/testing arena (40x40 cm) for 7 min each day. For training on the fifth day, mice were placed in the corner of the arena and allowed to explore for 7 min two identical objects (black 50 ml conical tubes filled with glass beads) placed along the midpoint of each opposite wall. Twenty-four hours after the training session (day 6), the mice were placed in the arena with one familiar object (black 50 ml conical from the training session) and one novel object (clear 50 ml conical tube filled with glass beads), placed in exactly the same locations as the two familiar objects from the training. The time spent investigating the familiar or novel objects was scored from video records by a blinded observer with a discrimination index calculated as: [time exploring novel object/(time spent exploring novel + time spent exploring familiar)]*100 [19]. This test was performed on 5 male MitoPark mice and 10 male wild-type littermate controls at 24 weeks of age.

Tail Suspension: To assess depressive or anti-depressive behavior, mice were suspended by the tail with tape for 6 min while a digital video camera recorded, and the total duration of immobility was counted [32]. Tail Suspension was performed on 6 male MitoPark mice and 10 male wild-type littermate controls at 26 weeks of age.

### Statistical analysis

Statistical analysis was performed using the R statistical language (Vienna, Austria). Data were tested for normality using Shapiro Wilk’s test. Group comparisons between MitoPark and controls were made using t-tests for normally distributed data and Mann-Whitney test for data which was not normally distributed in both groups. Data which showed significant deviation from normality (P < 0.05, Shapiro Wilk’s test), included whole brain volume and ADC of the sensory cortex in wild-type mice, and T2*, FA, and ADC of the substantia nigra in MitoPark mice. Correction for multiple comparisons was performed using the Benjamini and Hochberg false discovery rate [33] at a level of q = 0.05 for significant discoveries. Results are represented as mean ± standard deviation (SD), unless stated otherwise.

## Results

### Physiological parameters

Respiration rate (113–150 bpm), heart rate (403–580 bpm) and arterial oxygen saturation (93%-99%) of all animals were within normal physiological ranges and there were no significant differences in these physiological parameters between the two animal groups (p > 0.05). Body weight, an index of general health and vitality, of the control mice was 29.8±2.3 g at 30 weeks of age, and of the age-matched MitoPark mice was 19.2±2.6 g, indicating a 35.5% reduction (p < 0.05). The MitoPark mice, compared to controls, exhibited abnormal gait, reduced self-grooming, and significant loss of body weight, but were able to meet their daily needs (i.e., feeding and drinking) unassisted and to perform the behavioral tests.

### MRI findings

**Fig 1:**
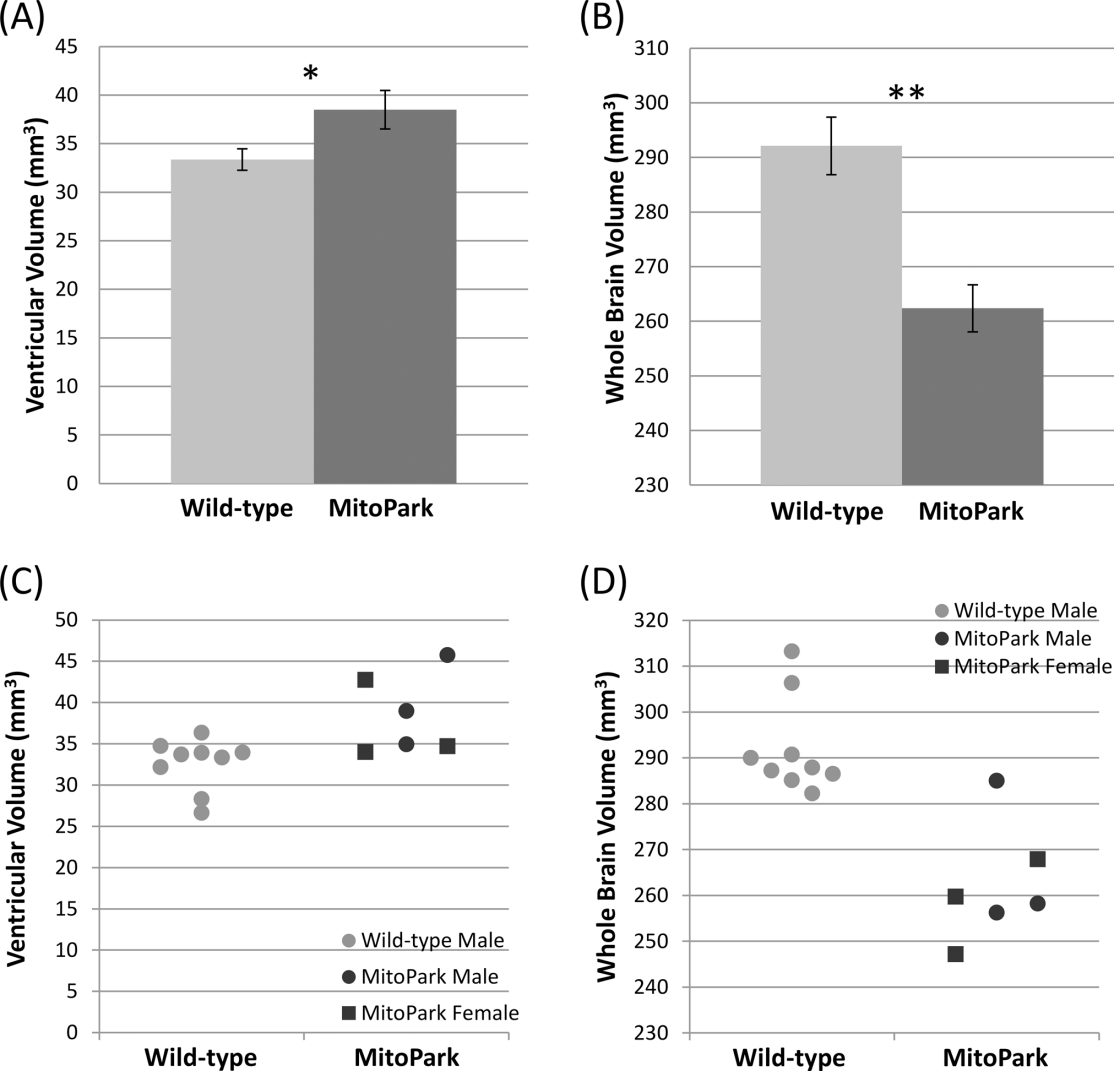
Group-averaged **(A)** ventricle and **(B)** whole-brain volumes of wild-type (n = 9) and MitoPark (n = 6) mice at 30 weeks of age. Mean ± SEM. * p < 0.05; ** p < 0.005 vs. wild-types. Scatter plots of individual **(C)** ventricle and **(D)** whole-brain volumes of male and female wild-type and MitoPark mice.

MitoPark mice showed enlarged lateral ventricles and reduced brain volume compared with control mice at 30 weeks of age (Fig 1). The group-averaged ventricular volume of MitoPark mice (38.5±4.9 mm^3^) was larger by 15% compared with control mice (33.4±2.7 mm^3^, p = 0.012). The whole-brain volume (excluding ventricular volume) of MitoPark mice (262±12 mm^3^) was smaller by 10% compared with wild-type mice (292±10 mm^3^, p < 0.001), suggesting brain tissue atrophy. There were not obvious differences in brain volume between male and female MitoPark mice relative to the male wild-type mice as shown in Fig 1C and 1D.

**Fig 2:**
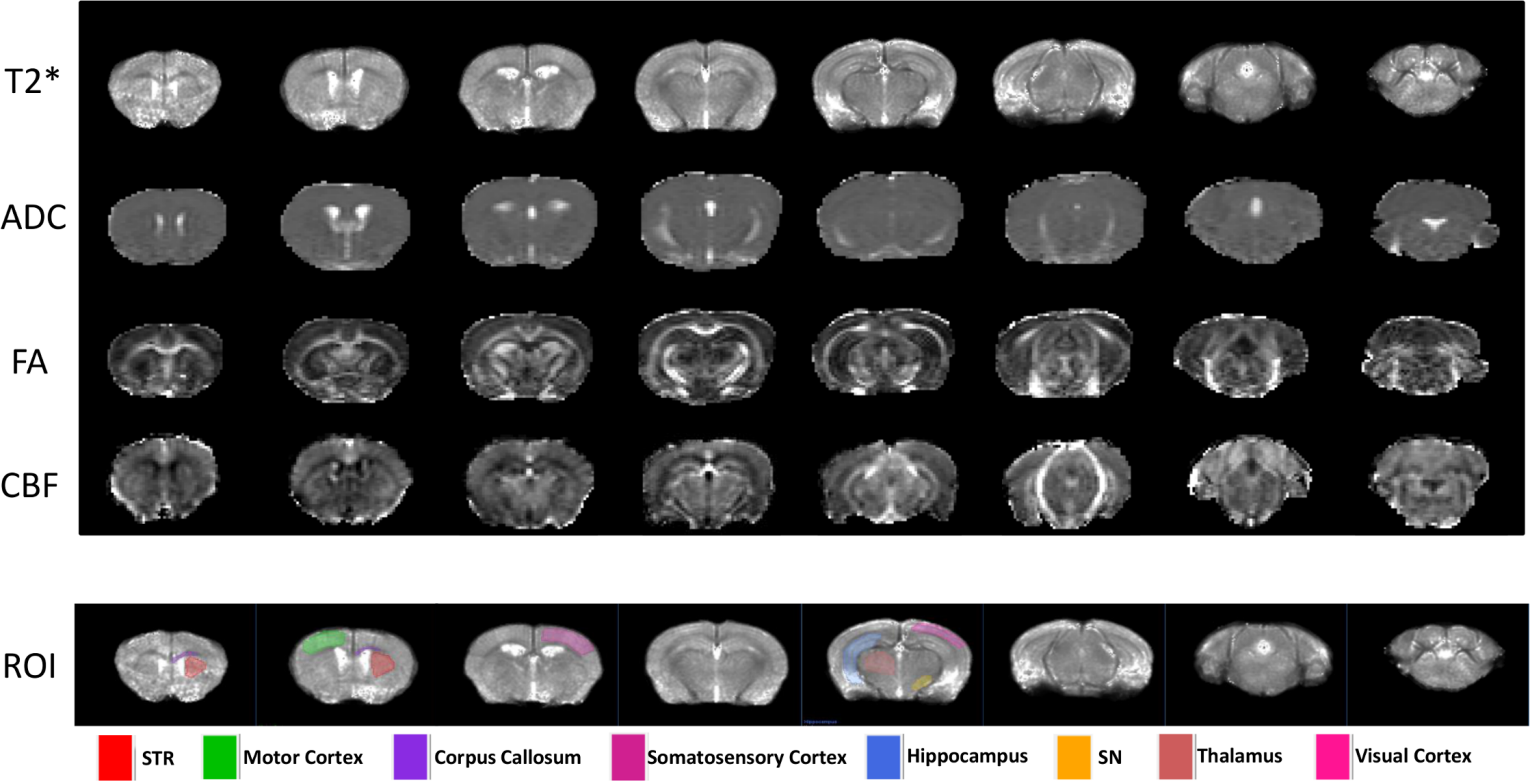
T2* maps, apparent diffusion coefficient (ADC), fractional anisotropy (FA) and cerebral blood flow (CBF) from one wild-type mouse, and ROIs of various brain regions used for quantitative analysis. Only unilateral ROIs are shown here, but bilateral ROIs were used for analysis. STR: striatum; SN: substantia nigra.

**Fig 3:**
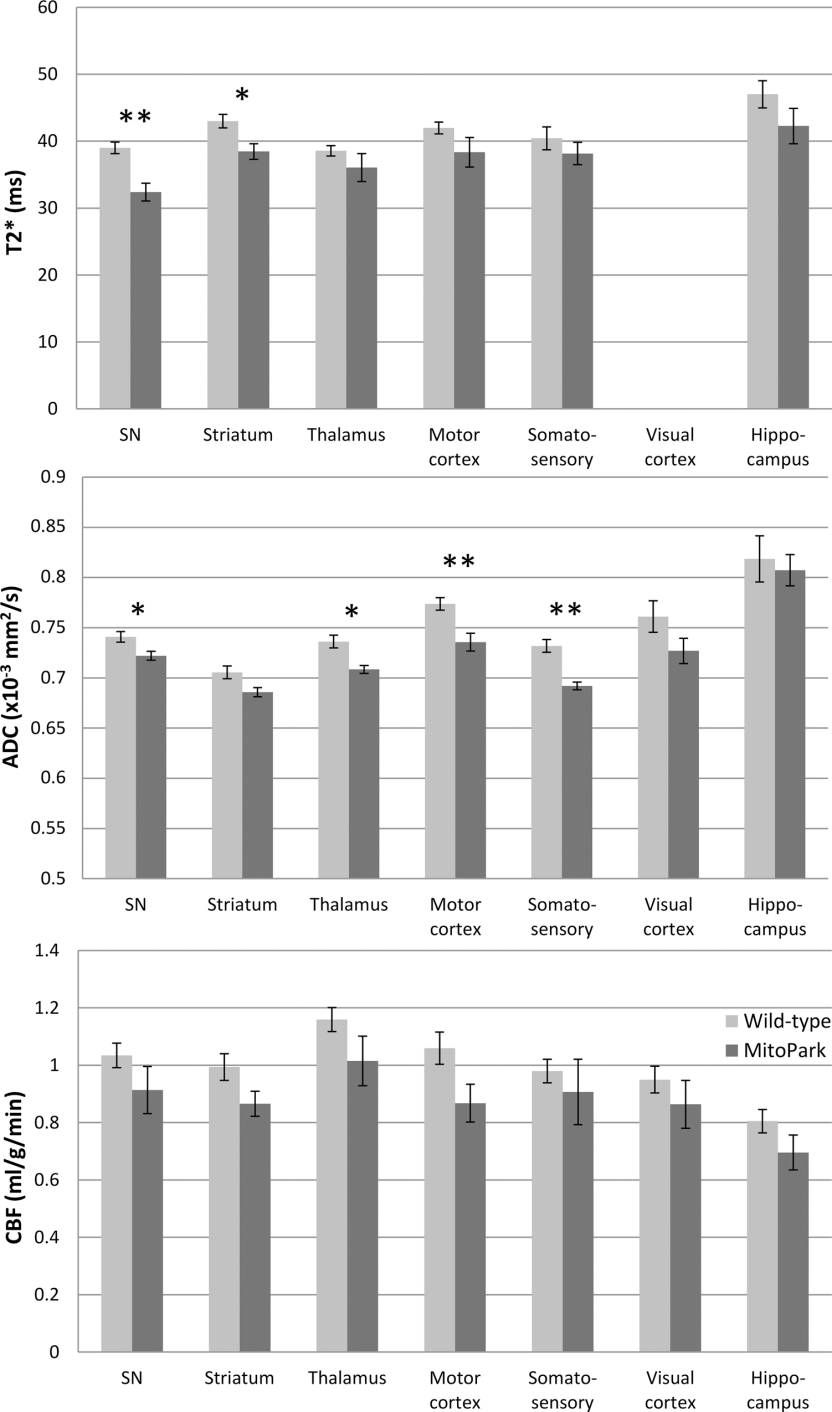
T2*, apparent diffusion coefficient (ADC), and cerebral blood flow (CBF) of the wild-type and MitoPark groups. Mean ± SEM. *p < 0.05; **p < 0.005 significant differences vs. age-matched wild-types.

Fig 2 shows representative T2*, ADC, FA, and CBF maps from a control mouse, along with the ROIs used for quantitative analysis. Group-averaged regional T2*, ADC and CBF values from MitoPark and controls are given in Table 1 and Fig 3. T2* values of the MitoPark group showed significant reductions in the striatum (p = 0.015) and substantia nigra (p = 0.004) compared with controls, consistent with iron deposition. There were no significant differences in T2* values in other brain regions analyzed. ADC values of the MitoPark group showed significant reductions in the substantia nigra (p = 0.012), motor cortex (p = 0.003), thalamus (p = 0.006), and sensory cortex (p < 0.001) compared with the control group. The ADC of the striatum also tended to be reduced, although not significantly (p = 0.042). There were no significant differences in ADC in the hippocampus or auditory-visual cortex (p > 0.1). Although slightly lower in all regions of MitoPark mice, CBF was not significantly different in any regions. CBF tended to be lower in the motor cortex (p = 0.046) but not significantly.

**Table 1:** T2*, apparent diffusion coefficient (ADC), and cerebral blood flow (CBF) of the wild-type and MitoPark groups.

	T2* (ms)		ADC (10^−3^ mm^2^/s)		CBF (ml/g/min)	
	Wild-type	MitoPark	Wild-type	MitoPark	Wild-type	MitoPark
	n = 5–6 ^a^	n = 4–5 ^a^	n = 9	n = 6	n = 9	n = 6
**SN**	**39.0 ± 2.11**	32.4 ± 2.98**	**0.741 ± 0.016**	0.722 ± 0.011*	1.03 ± 0.13	0.91 ± 0.20
**Striatum**	**43.0 ± 2.43**	38.5 ± 2.59*	0.706 ± 0.019	0.686 ± 0.011	0.99 ± 0.14	0.87 ± 0.11
**Thalamus**	38.6 ± 1.89	36.1 ± 4.18	**0.736 ± 0.019**	0.708 ± 0.010*	1.16 ± 0.13	1.01 ± 0.21
**Motor cortex**	42.0 ±1.96	38.4 ± 4.42	**0.774 ± 0.019**	0.736 ± 0.022**	1.06 ± 0.17	0.87 ± 0.16
**Somatosensory**	40.4 ± 3.80	38.2 ± 3.75	**0.732 ± 0.019**	0.692 ± 0.009**	0.98 ± 0.12	0.91 ± 0.28
**Visual cortex**	-	-	0.761 ± 0.047	0.727 ± 0.031	0.95 ± 0.14	0.86 ± 0.20
**Hippocampus**	47.0 ± 4.54	42.3 ± 5.29	0.818 ± 0.069	0.807 ± 0.038	0.81 ± 0.12	0.70 ± 0.15

^a^ sample sizes were smaller due to T2* image artifacts in some animals.

Mean±SD, significant differences are in bold with *p < 0.05; **p < 0.005 vs. age-matched wild-types.

**Fig 4:**
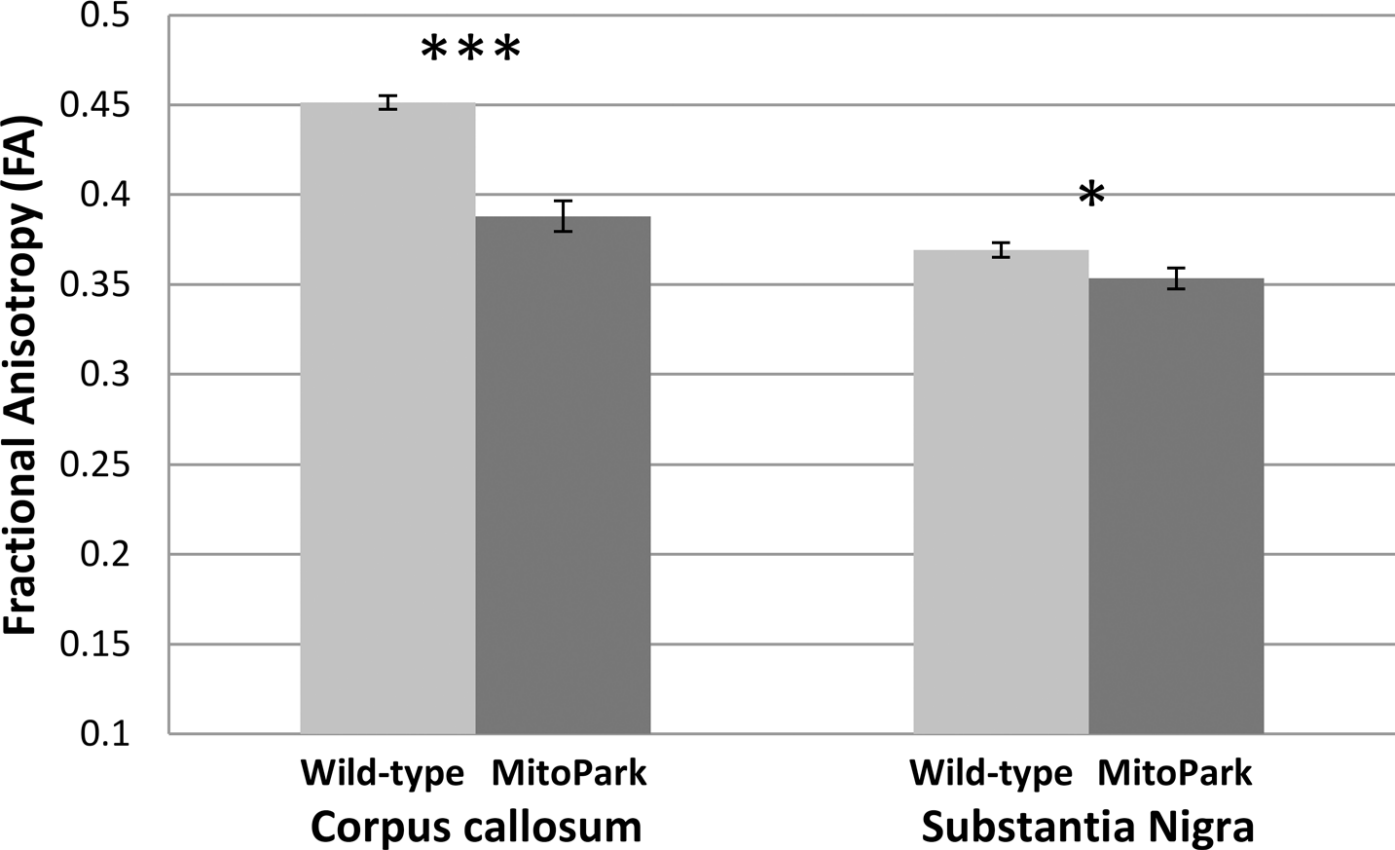
Fractional anisotropy (FA) of the corpus callosum (CC) and substantia nigra (SN) of the wild-type (n = 9) and MitoPark (n = 6) mice at 30 weeks of age. Mean ± SEM. * p < 0.05; *** p < 0.001 vs. wild-types.

FA in the corpus callosum (p = 4E-6) and substantia nigra (p = 0.036) of the MitoPark group was significantly reduced compared with the control mice (Fig 4). The high FA of the SN is consistent with studies which report FA values of ~0.5 in humans [34, 35] and 0.35 in mice [36], although it is possible that there could be some partial volume effects from the adjacent cerebral peduncles due to the small size of the SN.

### Behavioral findings

**Fig 5:**
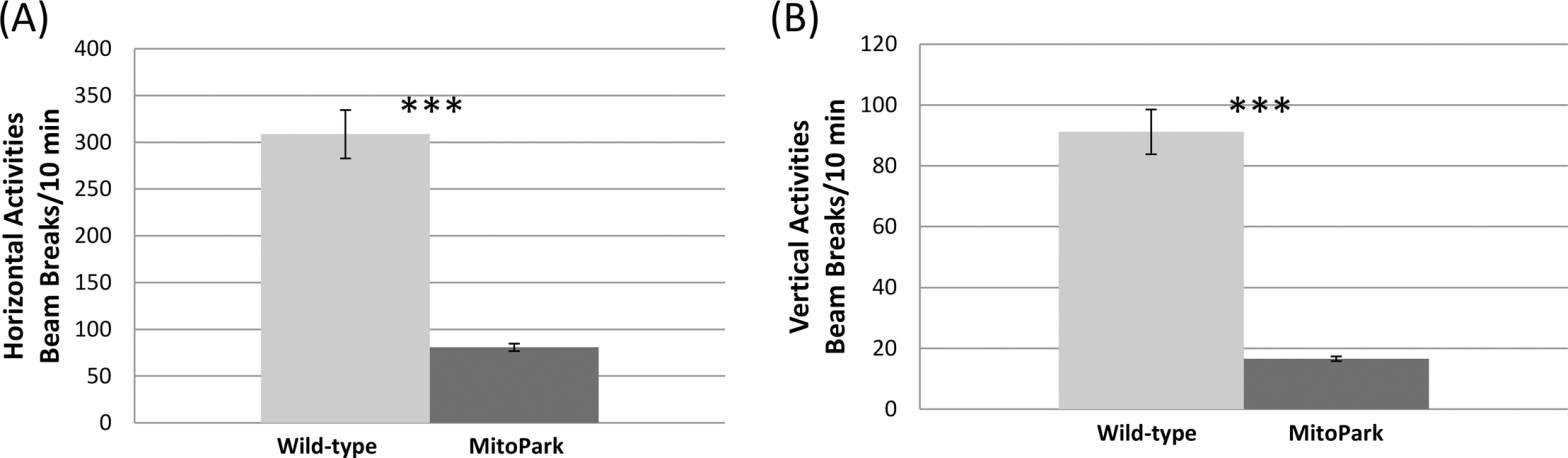
**(A)** Horizontal and **(B)** vertical locomotor activities for MitoPark (n = 8) and wild-type mice (n = 13) at 30 weeks of age. Mean ± SEM. *** p < 0.001 vs. wild-types.

**Fig 6:**
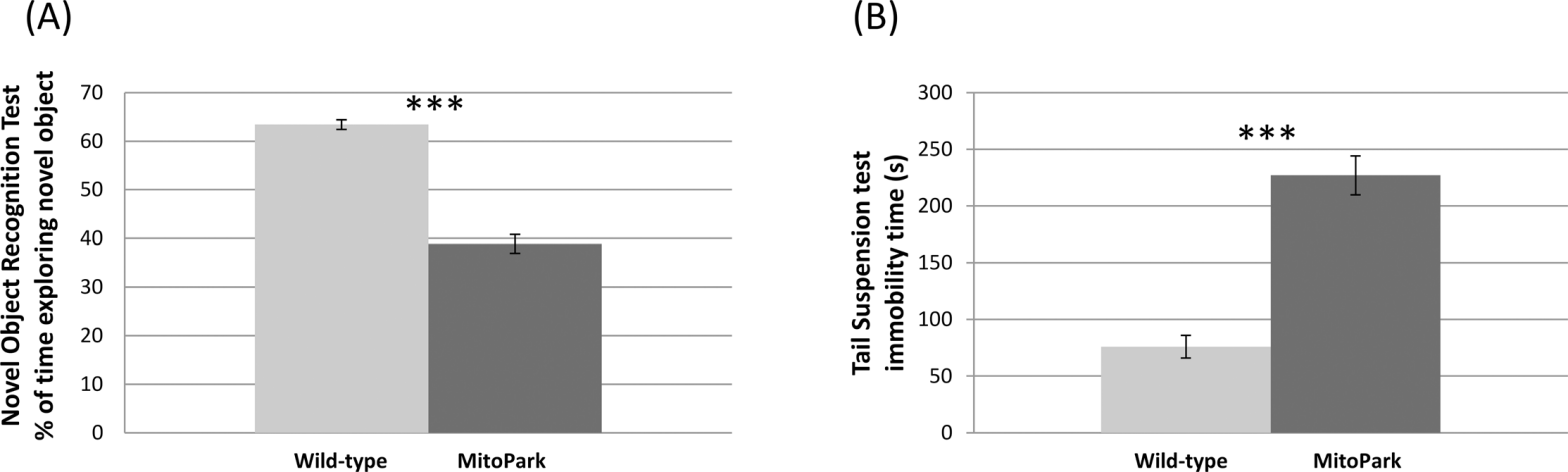
**(A)** Novel object recognition test for MitoPark (n = 5) and wild-type mice (n = 10) and **(B)** tail suspension test results for MitoPark (n = 6) and wild-type mice (n = 10) at 24–26 weeks of age. Mean ± SEM. *** p < 0.001 vs. wild-types.

General activity levels assessed by the open-field test demonstrated that, relative to control mice, MitoPark mice exhibited significantly reduced horizontal and vertical motor activities (p < 0.05; Fig 5). In the novel object recognition test, which assesses cognitive function, MitoPark mice spent significantly less time exploring the novel object (p < 0.05; Fig 6A). MitoPark mice also exhibited longer periods of immobility, characteristic of a depressive-like state, during tail suspension test (p < 0.05; Fig 6B).

## Discussion

Multi-modal MRI in the MitoPark mouse model of Parkinson’s disease detected abnormalities in the nigrostriatal and basal ganglia dopamine center, as well as widespread changes beyond the well-known dopamine circuits involved in PD pathology. Compared with age-matched controls, MitoPark mice at 30 weeks showed: i) increased ventricular volume and reduced whole-brain volume, ii) reduced T2* values in the substantia nigra and striatum but not in other brain regions, iii) reduced ADC in most brain regions analyzed, iv) reduced FA in the corpus callosum and substantia nigra, indicative of axonal damage, and v) reduced motor activity in open-field tests, reduced memory in novel object recognition tests, and signs of depression from increased immobility in tail suspension tests. In sum, MitoPark mice showed significant changes in multiple MRI parameters measured in the substantia nigra and striatum, some significant changes in different neocortical structures, but no significant changes in the hippocampus.

### Brain atrophy

MitoPark mice at 30 weeks of age showed significant brain atrophy, indicated by increased ventricular volume and reduced whole-brain volume. Increased ventricular volume and decreased brain volumes of several brain structures have been reported in a rat proteasome-inhibitor model of PD [37]. Human PD studies widely reported brain atrophy in many brain regions such as SN, striatum, and cortex [14–17]. Cortical atrophy is more severe in those PD patients who have dementia [16, 38]. Such brain atrophy likely arises from global neurodegeneration, including that of the dopaminergic system, and is associated with motor deficits and cognitive impairment.

### Increased iron accumulation

MitoPark mice showed T2* reduction in the striatum and substantia nigra, which is generally considered to be due to iron accumulation, but no other brain regions analyzed. Human studies reported consistent increases in iron deposition in the substantia nigra of PD patients [22, 39]. Iron deposition in the caudate-putamen of human PD patients is however variable, with different studies reporting higher [40], lower [41], or no changes in iron deposition [39, 42]. Increased iron accumulation in the substantia nigra is a consistent feature of both sporadic and familial forms of PD, and may provide a target for disease-modifying therapies. T2* MRI offers an alternative means for longitudinal monitoring of tissue iron deposits in vivo besides histology, although other underlying changes could also affect T2*. Studies are needed to verify independently the abnormal iron deposition in MitoPark mice in the striatum and substantia nigra.

### Neuronal degeneration detected by ADC

ADC in the SN of MitoPark mice was reduced. Indeed, by 30 weeks of age, MitoPark mice have lost approximately 80% of dopaminergic neurons in the substantia nigra, and the dopamine level in the striatum is only about 5% of that in age-matched control animals [12, 23]. Mueggler et al. reported reduced ADC in the cortex of amyloid overexpressing mice, which they suggested could be due to gliosis which was present in tissues with decreased ADC [43]. Gliosis is prominent in PD as well as Alzheimer’s disease [44]. Increased glial cells and processes and glial hypertrophy could impede diffusion in the extracellular space. There are likely multiple pathological microstructural changes (e.g. gliosis, lost neurons being replaced with interstitial fluid) occurring, which could have opposing effects on ADC. The significance of these different processes may also be dissimilar between models or stages of disease. By contrast, increased ADC values are reported in the substantia nigra in the MPTP toxin-based mouse model of PD wherein MPTP was administered intraperitoneally [36]. This discrepancy could be due to different types of neurodegeneration or severity of injury in different PD animal models.

We also found reduced ADC in other brain regions in MitoPark mice, including the somatosensory-motor cortex, consistent with the loss of neocortical dopamine cells reported in MitoPark mice [10]. We did not find significant ADC differences between MitoPark and age-matched controls in the hippocampus, consistent with a previous study that did not find changes in the hippocampus of MitoPark as indicated by normal dopamine levels [10]. Data on ADC changes in PD patients are variable. While many studies detected no ADC changes in the brain [19, 34, 45], some reported ADC increases in the striatum and thalamus [46], and one study reported ADC increases in some regions and decreases in others [47].

### Neuronal and white matter degeneration detected by FA

MitoPark mice showed reduced FA values in the SN and corpus callosum, suggesting neuronal and white matter degeneration, respectively. A number of studies have reported reduced FA in the substantia nigra of PD patients [19, 34, 35]. Reduced FA in the SN has been correlated with loss of SN dopamine neurons in MPTP mice [36]. Previous human PD studies also found reduced FA in the corpus callosum [34, 48]. The loss of white matter integrity is likely secondary to primary PD neurodegeneration.

### Blood flow

Basal CBF is tightly coupled to tissue metabolism. However, we only observed a non-significant slight reduction of CBF. In PD patients, there are heterogeneous CBF reductions in the caudate, temporal area, frontal area, and parieto-occipital cortex [20, 21], whereas CBF in the supplementary motor and primary sensorimotor cortex was unaffected [20, 21, 49, 50]. The differences in CBF findings between MitoPark mice and human PD could be due to different severity of the disease and/or different animal and human pathophysiology. Mitochondrial dysfunction in MitoPark mice may primarily affect the dopaminergic system, whereas multiple neurotransmitter systems in PD patients are generally affected, including cholinergic, serotoninergic, and noradrenergic systems besides the dopaminergic system.

### Functional (behavioral) changes

MitoPark mice exhibited significant motor deficits indicated by reduced horizontal and vertical locomotor activities. These findings are consistent with previous reports that MitoPark mice showed overt Parkinsonism-like hypokinesia at 30 weeks of age [12]. At this age, DA neurons in the substantia nigra are reduced to fewer than 20% that of controls, whereas, dopamine levels in the striatum are reduced to 5% that of controls. This stage is in line with the advanced PD stage in human [12, 23]. The motor deficits observed in the MitoPark mice are consistent with global brain atrophy and the quantitative MRI changes detected in the substantia nigra and striatum.

Although PD is primarily considered a movement disorder, non-motor components, such as cognitive impairment and affective disturbances, have profound negative impacts on the quality of life of PD patients. Indeed, at 30 weeks of age, MitoPark mice exhibited significant cognitive decline in novel object recognition test, and signs of depression-like state indicated by increased immobility time in tail suspension test. Note that severity of cognitive decline and signs of depression-like state could be confounded by the significant motor deficits that MitoPark mice exhibit at 30 weeks of age. Nevertheless, previous studies have reported cognitive decline in MitoPark mice prior to the development of motor dysfunction [11]. Finally, while we found reduced memory in novel object recognition tests, none of the MRI parameters surprisingly showed significant changes in the hippocampus which is important for visual object recognition memory [51]. Hippocampal atrophy has been reported in PD patients [52], while the only reported information we are aware of on the hippocampus in MitoPark mice is that dopamine levels remain normal at 20 weeks of age [10]. It could thus be that the hippocampus is unaffected in MitoPark mice, and other brain regions involved with the behavioral tests or hippocampal memory circuit, such as the perirhinal and entorhinal cortices [11], are affected. Alternatively, previous studies on MitoPark mice have found electrophysiological [53] and functional deficits [11] which occur as early as 6 weeks of age, which suggests that death of dopamine cells is not necessary to drive functional deficits; rather, a progressive loss in neuronal function that precedes cell death, but is not evident in anatomical MRI, may be sufficient. It is also possible that dopaminergic cells from the substantia nigra could modulate cortical and/or hippocampal processes indirectly via innervations of the thalamus. Further work is needed to better define the neuroanatomical correlates of cognitive dysfunction in MitoPark mice.

### Limitations

A limitation of this study is that data included both males and females because of small sample sizes. A previous study showed no interaction between sex and genotype on behavioral phenotypes [10, 11], suggesting the disease progresses similarly in both males and females. With our limited sample sizes there were not obvious structural differences between male and female MitoPark mice. Male C57BL/6J mice reportedly have 2.5% larger brains compared to females [54]. The brain volume of male wild-type C57BL/6J mice was 11.3% larger than MitoPark mice herein, which is notably larger than expected due to sex differences. Some previous MRI morphometry studies of mouse models of neurological disease found that sex was not a significant factor on brain structure [55, 56], but further studies with larger samples are needed to determine the effects of sex on MitoPark brain structure. Another limitation is that MRI and behavioral data were acquired on different groups of animals, which precluded quantitative correlation analysis. Finally, we did not tabulate the brain volumes of other smaller structures nor use voxel-wise analysis. ROI analysis was used to obtain quantitative MRI parameters for major brain structures. Future studies utilizing only the MRI protocols which are most likely to detect notable differences, which we found were diffusion tensor and susceptibility imaging in MitoPark mice, could use higher resolution which would allow for more refined regional analysis and registration between mice. Future studies will include longitudinal evaluation to assess parameters which may be useful to predict further progression, investigations of earlier stages of the disease, gender differences, validation of increased iron deposition by immunochemistry, histological correlations, and evaluation of novel therapeutic interventions.

## Conclusions

In conclusion, this study demonstrated that MitoPark mice recapitulate changes in many MRI parameters reported in PD patients. These MRI parameters have the potential to elucidate the temporal relationship of the onset and progression of neurodegeneration in the nigral dopaminergic and other systems in MitoPark mice. These tools may also be useful for longitudinal evaluation of novel therapeutics in animal models.

## Supporting Information

S1 Dataset Animal weights, Figs, and Tables. (XLS)
